# Are oral health and fixed orthodontic appliances associated with sports injuries and postural stability in elite junior male soccer players?

**DOI:** 10.1186/s13102-018-0105-5

**Published:** 2018-10-20

**Authors:** Henny Solleveld, John Flutter, Arnold Goedhart, Luc VandenBossche

**Affiliations:** 1 SportsInjuryLab, PO Box 3141, 3760 DC Soest, Netherlands; 2 General Dentist, 117 Warry Street, Fortitude Valley, 4006 Australia; 3 Physical Rehabilitation and Sports Medicine, Ghent University Hospital, De Pintelaan 185, 9000 Ghent, Belgium

**Keywords:** Oral health, Orthodontic appliances, Sports injury, Postural stability, Dental caries, Soccer

## Abstract

**Background:**

Dental caries and periodontitis are associated with elevated levels of pro-inflammatory cytokines which may trigger muscle fatigue during exercise, a strong risk factor for sports injuries. Fixed orthodontic appliances (FOA) may cause poor oral health and may disturb proprioceptive inputs of the stomatognathic system. This study aims to explore associations of poor oral health and of use of a FOA with injury frequency and postural stability.

**Methods:**

One hundred eighty seven Belgian elite junior male soccer players, aged 12–17 years, completed a self-report questionnaire asking about injuries in the past year, oral health problems, use of a FOA, demographics and sports data, and stood in unipedal stance with eyes closed on a force plate to assess postural stability.

**Results:**

Ordinal logistic regression with number of injuries in the past year as ordinal dependent variable and dental caries and/or gum problems, age and player position as covariates, showed that participants who reported dental caries and/or gum problems and never had had a FOA reported significant more injuries in the past year compared to the reference group of participants who reported no oral health problems and never had had a FOA (adjusted OR = 2.45; 95% CI, 1.19–5.05; *p* = 0.015). A 2 (temporomandibular joint problems) × 2 (FOA) × 2 (age) ANOVA with postural stabilities as dependent variables, showed a significant FOA x age interaction for the non-dominant (standing) leg. Post-hoc t-tests showed a significant better postural stability for the non-dominant leg (and a trend for the dominant leg) for the older compared with the younger participants in the non-FOA group (*p* = .002, ES = 0.61), while no age differences were found in the FOA-group.

**Conclusions:**

These results indicate that poor oral health may be an injury risk factor and that a FOA may hinder the development of body postural stability.

## Background

The injury rate of elite junior male soccer players is substantial, with an average of 1.3 [1] up to 1,75 [2] injuries per player per season. The main intrinsic risk factors for sports injuries in male, adolescent soccer players identified in a systematic review were: periods of accelerated growth and maturation, deficits in neuromuscular control, fatigue and previous injury [3]. Player position may be an extrinsic risk factor as Carling et al. [4] found that strikers had a significantly higher risk of injuries. Despite the common occurrence of poor oral health in athletes [5], possibly due to frequent use of acidic sports drinks and decreased salivary flow rate caused by mouth-breathing during heavy exercise [6], little research has examined its role in sports injuries [7, 8].

Periodontitis (gum problems) and dental caries, two aspects of poor oral health, may play a role in sports injuries because of their association with elevated levels of pro-inflammatory cytokines, like tumour necrosis factor (TNF-a) and interleukin-6 (IL-6) [9, 10]. These cytokines play an important role in the origin of muscle fatigue during exercise [11, 12], which is a main risk factor for sports injury [3].

As the dietary habits and oral hygiene of junior athletes using a Fixed Orthodontic Appliance (FOA) are closely monitored by the orthodontist, the high incidence of oral health problems that has been found in adolescents using a FOA [11], is most likely due to the orthodontic tooth movement that induces inflammation in the periodontal ligament and to the difficult-to-clean regions surrounding the brackets where cariogenic bacteria like Streptococcus mutans and Lactobacillus spp. can adhere and form a biofilm [13, 14]. Ghijselings et al. [15] found even 2 years after the use of a FOA more bleeding on probing in comparison with the baseline (pre-FOA) results, indicating more periodontal disease and, consequently, long lasting elevated levels of pro-inflammatory cytokines [9, 10]. This increase in bleeding on probing may be explained by the biofilm trapped by the wires bonded to the teeth to maintain the improved tooth position achieved by the FOA. Almost all FOA are followed by life time retention to maintain the improved tooth position.

Postural stability, an aspect of neuromuscular control, is not only a protective factor against injuries, but also crucial for sport success [16, 17]. The coordinated movement to keep proper postural stability, requires continuous integration of visual, vestibular and proprioceptive inputs [18]. As the proprioceptive inputs of the stomatognathic system (a functional unit that comprises the oral cavity, the temporomandibular joint (TMJ) and masticatory muscles) are processed in tandem with information from the vestibular and oculomotor systems [19], disturbances of the sensory information from the stomatognathic system may negatively affect postural stability. There is, however, mixed evidence on the impact of TMJ problems, another aspect of poor oral health, on postural stability [20–22].

Another possible influence of the use of a FOA on postural stability is due to the continuous mechanical force to teeth. This force may cause local tissue injury, which leads to the release of mediators like substance P and bradykinin from inflammatory cells [14, 23, 24]. These mediators stimulate the nociceptors of periodontal ligaments, thereby distorting the proprioceptive input from the stomatognathic system that is processed in tandem with information from the vestibular and oculomotor systems [19].

This study aims to examine the possible impact of poor oral health and the (past) use of a FOA on injury frequency and postural stability of junior elite soccer players. We specifically addressed the following research questions: (1) Are there associations of injury frequency during past year with gum problems and/or dental caries (aspects of poor oral health) and with current or past use of a FOA? (2) Is postural stability associated with current or past use of a FOA and with TMJ problems? We expect a higher frequency of sports injuries in the past year for players with gum problems and/or dental caries and, possibly to a lesser extent because of control of their oral health by the orthodontist, for players with current or past use of a FOA. With regard to postural stability, we expect a somewhat lower postural stability in players with current or past use of a FOA and in players with TMJ problems.

## Methods

### Procedure and participants

Four Belgian clubs in the highest professional soccer divisions were contacted and agreed to participate with their elite, junior male squads. The clubs were visited by the first author to further explain the aims and procedures of the project, to gather information on the number of players and to make appointments. Players and their legal guardians received a written description of the research procedure and informed consent and assent forms from their fully-informed team leaders.

Parental informed consent and adolescent informed assent was obtained for 187 participants, aged 12–17 years. Participants arrived forty-five minutes before training at the club, where they completed a questionnaire (detailing age, player position, past or present FOA, oral health and previous sport injuries) and then performed single-limb-standing balance tests, with and without cotton rolls between their premolars and first molar. The Dutch questionnaire was professionally translated into French and then translated back into Dutch to ensure accuracy. Ethical approval for this study was provided by the Ethical Committee of Ghent University Hospital.

### Questionnaire variables

*Oral health* (see Locker [25]) was assessed by 5 dichotomously scored questions on two kinds of oral health problems. *Gum Problems and/or Dental Caries (GP/DC)* were assessed with one question on gum problems: (1) “Do you have or have you had gum problems (bleeding, swelling or recession)?”, and with two questions on caries treatment that have been found valid as indicator of caries problems in previous research [26]: (2) “How many teeth were treated with fillings?” and (3) “How many teeth have been extracted?”. *Temporomandibular joint (TMJ) problems* were assessed with the questions (1) “Do you have or have you had problems with your jaw joint: problems with opening your mouth, clicking sounds, pain, difficulties with eating?” and (2) “Are you clenching or grinding your teeth?”. The four response categories of the questions on gum and TMJ problems were: never (scored 0) and sometimes, often and very often (scored 1), the four response categories of the questions on caries treatment were: none (scored 0) and one, two or three and four or more (scored 1). The *GP/DC* sum-score was dichotomized into 0 (absent) if the sum-score equals 0 and 1 (present) if the sum-score is above 0. *TMJ problems* were scored as 0 (absent) if the three TMJ items were scored 0, else TMJ problems were scored as 1 (present).

*Three Oral Status (OS) Groups* were composed, based on the GP/DC sum-score and the presence of a FOA: (1) ‘Both GP/DC and FOA absent’: participants who didn’t report GP/DC and who never had had a FOA, (2) Current or past FOA’: participants with current or past FOA, (3) ‘GP/DC present FOA absent’: participants who reported GP/DC and who never had had a FOA We refrained from differentiating GP/DC absent and present within the FOA group because (1) the question “How many teeth have been extracted?” is not a valid indication of oral health in the FOA group because extraction of teeth is often part of orthodontic treatment, and (2) the periodontal pathogen levels vary during orthodontic treatment with fixed orthodontic appliances [27].

#### Injury Frequency in the Past Year (IFPY)

In this study we used the broader definition of injury in the consensus statement: “Any physical complaint sustained by a player that results from a soccer match or soccer training, irrespective of the need for medical attention or time loss from soccer activities” [28]. IFPY was assessed with questions about how often each of the following body parts were injured in the past year: groin, hamstring, quadriceps, Achilles tendon, knee, ankle and other parts of the body i.e. “Have you had a knee injury in the past year?”. Participants responded by using a four-point Likert scale: never = 0, once = 1, two or three times = 2, and four times or more = 3. IFPY was computed as the sum of the 7 items and recoded into four categories that correspond with the response categories on each question: 0 = sum score 0 (no injuries), 1 = sum score 1 (one injury); 2 = sum score 2 or 3 (two or three injuries); 3 = sum score 4 and higher (at least 4 injuries).

*Player Position* was dichotomized into striker versus non-striker (goalkeeper, defender and midfielder). *Age* was dichotomized at the median as 12 to 14 versus 15 to 17 years of age. *Dominant leg* was based on the answer on the question about which leg is used most when kicking.

### Postural stability

As many technical movements of soccer players, like kicking and dribbling, are performed in single-legged stance under unstable conditions [29], we measured the postural stability of the participants while they stood in single-legged (unipedal) stance. The participants closed their eyes to eliminate visual input and thus give more weight to input from the stomatognathic system. Two 20-s. balance trials were performed on each leg barefoot, first without then with cotton-rolls between the posterior teeth. Cotton rolls were used because they may reduce possible negative effects of malocclusion on postural stability [30]. Participants were instructed to stand upright and to sway as little as possible, with the testing foot in the center of the force platform, their arms hanging loosely by their sides and the contralateral hip and knee flexed to approximately 30°. No instructions were given on the position of the supporting limb e.g. subjects were allowed to flex the knees.

Postural stability was evaluated by the Centre Of (foot) Pressure (COP) sway path length (the sum of the accumulated COP displacements, in millimetres measured with the Footscan USB2-system version 7.7 (RS Scan International, Olen, Belgium) composed of a 50 × 40 cm foot pressure platform with 4096 sensors, sampling at 100 Hz, an USB interface box to connect to a personal computer and software to calculate the COP sway path length. The mean of the COP sway path lengths with and without cotton rolls was used as measure of postural stability on each leg.

### Statistical analysis

Outliers on each of the four balance trials (unipedal standing on each leg, with and without cotton rolls), were identified as values that exceed the upper quartile plus 1,5 times the interquartile range (the outlier cut-off value) and were replaced by the highest observed value below the outlier cut-off value [31]. The chi-square test was employed to evaluate whether use of a FOA was associated with background variables (e.g. age) and whether oral status (OS) was associated with the number of injuries in the past year (IFPY). If the association between OS and IFPY was significant, two sets of analyses were performed. First, adjusted standardized residuals (ASR’s) were used to determine which cells contributed to the significant finding: |ASR| > + 1,96 indicate a significant contribution [32]. Second, ordinal logistic regression was used to examine whether the association between number of injuries (the ordinal dependent variable) and oral status would persist after controlling for age and playing position (striker vs. non-striker). To decide upon the categorization of age in the ordinal logistic regression analysis, polynomial contrast analysis was used to test for linear, quadratic and cubic trends of age on number of injuries was performed. The Brant test was used to evaluate the parallel regression assumption of ordinal logistic regression, the fit of the regression model was assessed by the Pearson’s chi-squared goodness-of-fit test and Nagelkerke R^2^ was used to indicate the proportion of explained variation in the number of injuries. The association of COP sway path length of each leg as dependent variables, with FOA-use (past or current versus never), TMJ-problems (present versus absent) and age (12–14 versus 15–17 years) as independent variables, was tested by using 2 (FOA use) × 2 (TMJ-problems) × 2 (age-groups) Analyses of Variance (ANOVAs). Significant interaction effects were analysed by post hoc t-test. All tests were two-tailed, and a *P* value of less than 0.05 was considered to indicate statistical significance. Statistical analyses were conducted using S-Plus 2000 (Insightful Corp., Seattle, WA, USA).

## Results

The mean age of the study participants was 14.7 years (SD = 1.7). Table 1 summarizes the age categories, playing position, GP/DC, and TMJ problems of the sample, broken down by Oral Status. Current or past use of a FOA was more often found with older participants, but less often found among strikers. TMJ problems were most often found among the ‘GP/DC present FOA absent’-group. On further analyses we found a significant higher proportion of participants with one or more extracted teeth in the FOA group (40% vs. 17%; *p* < 0.001).

**Table 1:** Age, playing position, oral health problems and dominant leg by Oral Status

Characteristic	Oral Status			*p*-values^a^
	Both GP/DC and FOA absent N (%),	Current or past FOA N (%)	GP/DC present FOA absent N (%)	
Age				=0.001
12–14 years (0)	24 (50%)	24 (32%)	40 (62%0	
15–17 years (1)	24 (50%)	51 (68%)	24 (37%)	
Playing position				< 0.001
Non-striker (0)	30 (62%)	67 (89%)	37 (58%)	
Striker (1)	18 (37%)	8 (11%)	27 (42%)	
GP/DC				
Absent (0)	48 (100%)	21 (31%)	0 (0%)	
Present (1)	0 (0%)	52 (69%)	64 (100%)	
TMJ problems				=0.02
Absent (0)	38 (79%)	48 (64%)	34 (53%)	
Present (1)	10 (21%)	27 (36%)	30 (47%)	

*GP/DC* Gum problems and/or dental caries, *TMJ problems* Temporomandibular joint problems; ^a^chi-square test

### Injury frequency in the past year (IFPY)

A significant association was found between IFPY and oral status (χ^2^(6 *df*) = 19.87, *p* < 0.005). The adjusted standardized residuals (ASR) indicated four cells with a significant difference between observed and expected frequency (see Table 2). In the ‘GP/DC present FOA absent’-group we found a significantly lower proportion of players who reported no injuries in the past year (19%) and a significantly higher proportion of players who reported 2 or 3 injuries (45%). In the ‘Current or past FOA’- group a significantly lower proportion (23%) reported 2–3 injuries, a significantly higher proportion (16%) reported 4 or more injuries.

**Table 2:** Injury Frequency in the Past Year (IFPY) by Oral Status

Number of sport injuries past year	Oral Status		
	Both GP/DC and FOA absent N (%), ASR	Current or past FOA N (%), ASR	GP/DC present FOA absent, N (%), ASR
0	20 (42%) + 1.9	26 (35%) + 0.9	12 (19%) - 2.6
1.	13 (27%) - 0.3	20 (27%) - 0.5	21 (33%) + 0.9
2–3	13 (27%) - 0.8	17 (23%) - 2.1	29 (45%) + 2.9
4 or more	2 (4%) - 1.3	12 (16%) + 3.0	2 (3%) - 1.9

*ASR* Adjusted Standardized Residual (|ASR| > 1,96 indicates cell with observed frequency significant different from the expected frequency); *GP/DC* Gum problems and/or dental caries; *FOA* Fixed Orthodontic Appliance; IFPY is significantly associated with oral status (χ^2^(6 *df*) = 19.9, *p* < .005)

Using polynomial contrast analysis, a linear trend of age on number of injuries was found (F(1,181) = 4.22, *p* = 0.04). To prevent a large number of cells with zero frequency in the ordinal logistic regression analysis, we used two age categories (12–14 versus 15–17 years old) and three ordinal categories (i.e. 0, 1 and 2 or more) of the number of sports injuries in the past year. The proportional odds assumption of the ordinal logistic regression analysis with number of injuries as (ordinal) dependent variable and oral status, age and player position as factors, was confirmed (χ^2^ (4 df) = 4.65, *p* = 0.32), Pearson’s chi-squared goodness-of-fit test indicated good fit of the model (χ^2^ (18 df) = 14.33, *p* = 0.71) and Nagelkerke R^2^ indicated that 11% of the variance of number of injuries was explained.The results of the ordinal logistic regression analysis confirmed that players of the ‘GP/DC present FOA absent’-group reported significant more injuries in the past year compared to players of the ‘GP/DC and FOA absent’-group (the reference category): adjusted OR = 2.45, *p* = 0.015, 95% CI 1.19–5.05. The number of injuries in the ‘Current or past FOA’ group was higher but not significantly different from the ‘GP/DC and FOA absent’ group (adjusted OR = 1.66, *p* = 0.163. 95% CI 0.81–3.40). The strikers reported much more injuries in the past year compared with the other players (adjusted OR = 2.44, *p* = 0.003, 95% CI 1.26–5.26), older players reported not significantly more injuries (adjusted OR = 1.59, *p* = 0.11, 95% CI 0.90–2.51).

### Postural stability

The 2 (non-FOA vs FOA) × 2 (TMJ problems absent vs present) × 2 (12–14 vs. 15–17 years) ANOVA with postural stability while standing on the dominant leg (shooting leg) as dependent variable, yielded no significant effects. The identical ANOVA with postural stability while standing on the non-dominant leg as dependent variable, yielded a significant interaction effect FOA group x age (F (1,178) =9.40, *p* = 0.003). Follow-up t-tests in the non-FOA group (see Table 3) indicated a better postural stability of the older participants compared with the younger participants while standing on the non-dominant leg (t(110) = 3,18, *p* = 0.002, ES = 0.61) and a trend for standing on the dominant leg (t(110) = 1,87, *p* = 0.068, ES = 0.36). As shown in Table 3, we didn’t find a better postural stability of the older participants of the FOA group compared with the younger ones.

**Table 3:** Means (standard deviations) of COP sway path lengths by FOA group, standing leg and age

FOA group	Standing leg	COP sway path lengths (mm)		*P*-value (ES)
		12–14 years M (SD)	15–17 years M (SD)	
Current or past FOA	Dominant leg	1240 (611)	1192 (485)	0.71 (0.09)
	Non-Dominant leg	1156 (424)	1298 (478)	0.22 (0.31)
Never used a FOA	Dominant leg	1396 (609)	1189 (536)	0.07 (0.36)
	Non-Dominant leg	1322 (425)	1079 (366)	0.002 (0.61)

Note: (Non-)Dominant leg = (non-)dominant leg for kicking the ball. Lower COP sway path lengths indicate better postural stability

## Discussion

The aim of the current study was to investigate two research questions concerning the relationship in junior elite soccer players of poor oral health and of use of a FOA to injury frequency and postural stability. The first research question sought to examine whether self-reported gum problems and/or dental caries (two aspects of poor oral health) and current or past use of a FOA were associated with higher injury frequency during past year. The second sought to explore associations between TMJ problems (an aspect of poor oral health) and current or past use of a FOA with postural stability.

Findings indicated a negative influence of poor oral health on injury frequency as significant more injuries in the past year were reported by participants who reported gum problems and/or dental caries and never had had a FOA. With regard to the second research question, we found indirect evidence for a negative role of a FOA in the development of postural stability in elite junior male soccer players as no progression of postural stability with age was found in the FOA group, while the older participants in the non-FOA group showed significant better postural stability compared with the younger ones when standing on the non-dominant leg, and a trend (*p* < 0.07) when standing on the dominant leg.

Associations of poor oral health with injury frequency were also found with adult elite soccer players [7, 8]. As elevated levels of pro-inflammatory cytokines are observed in poor oral health conditions, we would argue that these associations lend support to the prominent role of pro-inflammatory cytokines in the induction of muscle fatigue during exercise [11, 12, 33]. Muscle fatigue is a risk factor for sports injuries, as indicated by the increasing proportions of injury over time in the first and second halves of soccer matches [34], because muscle fatigue reduces the energy-absorbing capabilities, thereby increasing incorrect exchanges of proprioceptive information needed to maintain postural stability [3, 35]. Based on this line of reasoning, one might suggest that the findings on associations between poor oral health and sports injuries are also relevant for other teens who regularly perform vigorous or strenuous physical activities, like junior elite soccer players do.

Elevated levels of pro-inflammatory cytokines are not only found with dental caries and periodontitis. They are generally found with infections and inflammatory conditions and diseases like allergy, asthma, autoimmune diseases, hepatitis and coeliac disease. Therefore, one might expect that more sports injuries will also be found in elite soccer players with infections and inflammatory conditions and, more generally, in individuals with infections and inflammatory conditions who are involved in strenuous physical activities on a regular basis.

A complex result was obtained with regard to the association of past or present FOA with sports injuries in the past year. In the group with past or present FOA we found a slightly higher proportion of participants with zero injuries and a significantly lower proportion of participants with two or three injuries, but we also found a significantly higher proportion of participants with four or more injuries in the past year. This result may indicate that past or current use of a FOA as such is not associated with a higher frequency of injuries, but that there may be a subgroup of FOA-users with a high risk of sport injuries, for example a subgroup with pain-problems, like head- or neck-pain, as pain distort exchanges of proprioceptive information [19].

We found indirect evidence for a negative role of a FOA in the development of postural stability in elite junior male soccer players, but not for a negative role of TMJ-problems. These findings indicate that the development of better postural stability may be hindered by FOA-related distortions of the proprioceptive input from the stomatognathic system, like the release of mediators from inflammatory cells that stimulate the nociceptors of periodontal endings [23.24]. In addition, the forced change of the tooth position by using a FOA may also distort the proprioceptive input.

Several limitations of this study deserve consideration. First, the reliability of self-reports on injuries may be restricted due to response bias (i.e. denial of vulnerability), careless response and lack of insight. However, there are no reasons to expect differences in the reliabilities of self-reports on injury between the oral status groups, and vice versa. In addition, as lower reliability generally results in lower associations, the associations found in this study may be conservative. Second, self-reports on oral health are less reliable and less valid than clinical observations, which may have weakened our results. In particular, we may have underestimated the possible role of TMJ-problems. Third, the participants were not randomly selected from all elite junior male soccer players. They were recruited from Belgium’s highest division professional soccer clubs with a medical staff that had a positive attitude towards this study. Fourth, we examined only static postural stability and used only one COP parameter: the COP sway path length. While comparable results may be expected with equivalent static postural stability measures, the results may have been different if we had also used measures of dynamic postural stability and/or different testing conditions, e.g. including fatigue conditions. Finally, as a cross-sectional design was used, the results offer only preliminary evidence for the associations of Oral Status with sports injuries and postural stability. Hence, the results of this study should be confirmed in a prospective study.

## Conclusions

The current study has shown evidence that gum problems and/or dental caries are associated with increased sports injuries in elite junior male soccer players, even after controlling for age and striker vs non-striker position. As sports injuries may negatively affect the progression of the juniors and as oral health is an important part of general health and well-being, these results underline the need for oral health promotion and monitoring strategies. In addition, we found indirect indications that elite junior male soccer players with past or present FOA may need more neuromuscular training and extra guidance as we found indirect evidence for less development of postural stability in comparison with players without a FOA. Further, a subgroup of the FOA-users reported a very high number of sports injuries in the past year. More research on oral health and FOA use by athletes would be welcomed.

## Abbreviations

ASR: Adjusted Standardized Residual
COP: Centre Of foot Pressure
FOA: Fixed Orthodontic Appliance
GP/DC: Gum problems and/or dental caries
IFPY: Injury Frequency in the Past Year
TMJ: Temporomandibular Joint
